# Knowledge, attitudes, and practices of Lebanese patients with type II diabetes towards the use and abuse of dietary supplements: A cross-sectional study

**DOI:** 10.12688/f1000research.146998.2

**Published:** 2024-07-26

**Authors:** Maher Abdallah, Sahar Dandachy, Nour Ahmad, Marwa Sleiman, Rania Mansour, Maha Hoteit

**Affiliations:** 1 Faculty of Public Health, Section 1, Lebanese University, Beirut, Lebanon; 2 Faculty of Public Health, Section 3, Lebanese University, Tripoli, Lebanon; 3 School of Social Sciences and Humanities, Program of Social Work, Doha Institute for Graduate Studies, Doha, Doha, Qatar; 4 Food Science Unit, National Council for Scientific Research Lebanon (CNRS-L), Beirut, Lebanon

**Keywords:** Lebanon, type 2 diabetes, KAP, dietary supplements

## Abstract

**Background:**

Dietary supplements (DS) use among Lebanese patients with type 2 diabetes mellitus (T2DM) increased widely due to the country’s economic and financial situation. This study was conducted (1) to estimate the prevalence of DS use among persons with T2DM amid the escalating economic crisis in Lebanon; (2) to explore the knowledge, attitude, and practice (KAP) of DS use; and (3) to determine any significant association between socio-economic and socio-demographic factors and the use of DS modality

**Methods:**

A cross-sectional study was conducted during the worst episode of the economic crisis between October and April 2022 on 460 adult patients with T2DM of both sexes. Patients were interviewed using a pre-tested questionnaire.

**Results:**

Almost 4 out of 10 patients with T2DM in our study were found to be using DS, where 27.6% take multivitamins frequently. One-third of the participants agreed that nutritional supplements are necessary to control diabetes symptoms and complications. Around 41.1% of the participants complained about hypoglycemia and used DS to control their blood sugar levels (56.4%), while the rest used it to improve their health (35.5%) and control their diet (2.2%). The predictors of DS usage were the patient’s level of education [OR=3.9, CI=1.5-10, p=0.003), self-monitoring of blood sugars (OR=4.9, CI=1.68-14.6; p=0.004) and reading the nutrition label [OR=59.3, CI=6.3-55.8, p=0.000].

**Conclusion:**

This study estimated the prevalence of DS use and abuse, among persons with diabetes type II and found three significant predictors of DS use among patients with T2DM. Public health experts should encourage healthy discussions and awareness with their patients to comprehend their views regarding DS use.

**Abbreviations**
CAM: Complementary and alternative medicine
COVID-19: Coronavirus infectious disease
DS: Dietary supplements
GCC: Gulf Club Countries
KAP: Knowledge, attitude, and practice
LMICs: Low- and middle-income countries
NCDs: Non-communicable diseases
SDG: Sustainable developmental goals
T2DM: Type 2 diabetes mellitus
USA: United States of America

## 1. Introduction

Among the sustainable developmental goals (SDG), SDG 3.4 addresses Non-Communicable Diseases (NCDs) specifically, and world leaders committed to a one-third reduction in deaths between the ages 30 to 70 years from diabetes by the year 2030. Although the burden is worldwide, low- and middle-income countries (LMICs) are struggling with NCDs, especially Lebanon, with over three-quarters of all NCD deaths occurring in these countries. ^1^ In Lebanon, 91% of all deaths are attributed to NCDs, ^2^ and the initiatives, led by the Ministry of Public Health, to address the NCD burden failed to be implemented adequately. ^3^^–^^5^ This is due to the country’s political and economic challenges. ^6^ On the other hand, Lebanese patients with type 2 diabetes mellitus (T2DM) are experiencing medicine shortages ^7^ with rise in medication prices, which leads the patients to be incapable of buying their medical drugs. This deviation increased the health risk for these patients and increased the use and abuse of dietary supplements (DS). This increase may be due to several factors, including patients' perceptions that DS's natural products are safer, more effective, and cheaper than conventional medicines. ^8^ In Lebanon, there are no studies that investigate the prevalence of DS use and abuse among persons with T2DM or their knowledge, attitudes, and practices toward DS use. Our research group had already investigated DS use among children under 5 and their mothers (DS use: 64%), ^9^ among adults before versus during the COVID-19 pandemic (DS use: 73% versus 70%, respectively), ^10^ and among athletes (DS use: 74%). ^11^ On the other hand, in the United Sates, approximately 54% of individuals with diabetes used dietary supplements based on data from the National Health and Nutrition Examination Survey (NHANES) from 1999 to 2014. Older adults over 65 years had the highest usage at 62%, followed by those aged 40 to 64 years at 51%. Individuals aged 20 to 39 years had the lowest usage at 30%. Factors associated with higher dietary supplement consumption among individuals with diabetes included older age, female gender, non-Hispanic white ethnicity, higher education level, longer duration of diabetes, and presence of comorbidities such as cancer, chronic kidney disease, cardiovascular disease, and chronic obstructive pulmonary disease. ^12^ However, 6217 studies suggest limited clinical evidence supporting the use of dietary supplements for reducing or controlling diabetes. ^13^

At national level, conducting an assessment of knowledge, attitudes, and practices of dietary supplements among patients with Type II diabetes during the current Lebanese economic crisis is crucial for several reasons: 1) access to healthcare services and resources may be limited, leading individuals to seek alternative methods for managing their health conditions. 2) Understanding the knowledge, attitudes, and practices of dietary supplements among patients with Type II diabetes can help healthcare providers identify potential gaps in information and provide targeted education and support. 3) Individuals may experience heightened stress levels, changes in routine, and disruptions to their usual healthcare management. This can impact their dietary habits, medications use and supplement use, potentially leading to suboptimal health outcomes. By assessing the knowledge, attitudes, and practices of dietary supplements among patients with Type II diabetes, healthcare providers can tailor interventions to address specific needs and promote safe and effective supplement use during challenging times. Thus, the aims of the current study are: (1) to estimate the prevalence of DS use among persons with T2DM amid the escalating economic crisis in Lebanon; (2) to explore the knowledge, attitude, and practice (KAP) of DS use; and (3) to determine any significant association between socio-economic and socio-demographic factors and the use of DS modality.

## 2. Methods

### 2.1 Study design

A cross-sectional study was conducted between August 1 and November 2022 across the 4 main governorates in Lebanon (Beirut, Mount Lebanon, Beqaa, and North Lebanon). This study was conducted in accordance with the ethical principles outlined in the Declaration of Helsinki. After the approval of the ethical committee at al Zahraa University Medical Center (#157/May 7, 2022), we approached the medical files of patients with T2DM to retrieve their phone numbers from many hospitals, outpatient departments, and private clinics in Lebanon. Patients signed a written consent form before being enrolled in the study.

### 2.2 Sample

Convenience sampling was the method adopted to collect the information from the respondents. A total of 460 patients with T2DM were enrolled in this survey. The response rate was 100%.

***2.2.1 Inclusion and exclusion criteria***

The criteria for inclusion of patients in the current study were: participants with T2DM who are older than 18 years and have given their informed consent to take part in the study. The exclusion criteria were individuals under the age of 18 years because of the high probability of having type 1 diabetes mellitus at this age. Moreover, all older patients with diabetes type 1 were also excluded. In addition, pregnant women with gestational diabetes, elderly people, and people with intellectual disabilities were excluded from this study.

### 2.3 Questionnaire

Using a standardized questionnaire, a face-to-face interview between licensed dietitians and the patients was conducted. To ensure the validity of the data collection tool, the questionnaire was adapted from relevant literature in English, then translated by experts into Arabic and back-translated into English to check the translation. ^14^ The data was gathered using the administered questionnaire in Arabic. The questionnaire was made to find out the participants’ KAP on DS use as well as to identify the prevalence and most popular DS modalities in Lebanon. It was divided into five sections: (1) demographics, (2) clinical data about T2DM, (3) knowledge, (4) attitude, and (5) practice of DS therapies. All information about the patient’s demographics, including gender, age, marital status, level of education, and employment status, was recorded. Additionally, the time of the first time being diagnosed with T2DM, the type of T2DM therapy (insulin or oral medicines), the complications related to T2DM, and other co-morbid issues are clinical factors that were included in the study. In the knowledge section, we checked the knowledge of patients with T2DM with regard to DS efficacy and safety concerns. Moreover, in the attitude part, participants were asked about their attitudes toward the use of DS. Would they, for example, abide by their doctor’s recommendations to avoid using DS. Would they consult their physicians before using DS or not. In the practice section, we asked patients with T2DM if they had ever used DS for diabetes specifically. Participants who replied “yes” were then questioned about the type of DS used, who prescribed it, whether they were informed by their physician about it, whether they combined it with their T2DM medications, and whether they had ever used DS for a condition other than T2DM.

### 2.4 Data management and analysis

The data was coded and checked for completeness and consistency. All responses from the questionnaire were entered into Microsoft Excel, and a quality check was performed; data cleaning. Then it was exported to SPSS. Statistical analysis was conducted using SPSS (IBM Corp, SPSS Statistics version 26) ( https://www.ibm.com/support/pages/spss-statistics-260-fix-pack-1). SPSS was used for data entry, coding, data management, and analysis. The results were described as frequencies and percentages for variables. The associations between both the demographic factors and the clinical data about T2DM with DS were determined using Pearson’s Chi-square test. A *p value* of ≤ 0.05 was considered significant. Odds ratio was considered as a measure of strength. Multivariate logistic regression was used to identify predictors of DS usage.

## 3. Results

### 3.1 Sociodemographic Characteristics of the study population

Four hundred and sixty subjects participated in this study. The frequency distribution of their socio-demographic findings is presented in Table 1. Most of the participants were aged 41-60 years (51.3 %), males (51.3 %), from North Lebanon (49.1%), married (60.2%), had a university degree (39.8%), unemployed (41.3%), and had no monthly income (35.4%)

**Table 1.** Socio-demographic characteristics of the study participants (N=460).

Socio-demographic variable	N (%)
**Gender**	
Male	236 (51.3)
Female	224 (48.7)
**Marital status**	
Single	113 (24.6)
Married	277 (60.2)
Divorced	37 (8.0)
Widowed	33 (7.2)
**Age (years)**	
18-40	163 (51.3)
41-60	101 35.4)
61-65	61 (13.2)
**Education**	
Illiterate	76 (16.5)
Primary level	65 (14.1)
Secondary level	64 (13.9)
High school level	72 (15.7)
University level	183 (39.5)
**Employment**	
Don’t work	190 (41.3)
Private job	108 (23.5)
Part-time job	62 (13.5)
Full-time job	100 (21.7)
**Income**	
No income	163 (35.4)
less than 1.5 million lira Lebanese	26 (5.7)
between 1.5 and 3 million lira Lebanese	53 (11.5)
greater than 3 million lira Lebanese	36 (7.8)
less than 100 dollars	38 (8.3)
between 100 and 300 dollars	66 (14.3)
greater than 300 dollars	78 (17)
**Address**	
Beirut and Mount Lebanon	76 (16.5)
North Lebanon	352 (56.5)
Beqaa	32 (5.8)

### 3.2 Participant’s medical characteristics

The clinical data characteristics of the study participants with T2DM are shown in Table 2. Most of the patients conducted their laboratory tests three months prior to the data collection (37%), and around 34% were newly diagnosed with T2DM. Furthermore, approximately 32% of the study participants developed diabetic complications such as neuropathy (5.7%), nephropathy (5.7%), diabetic foot syndrome (7.2%), and retinal disease (13.5%), which was the most common. Most of the study participants had a family history of T2DM (88.7%), most of them inherited T2DM from their fathers (31.3%). As for medical treatment, the vast majority of patients (72%) were on oral anti-diabetic medications, and most of them had other medical comorbidities such as coronary artery disease (21.3%), osteoporosis (9.3%), kidney disease (8.7%), and hypertension (23.7%).

**Table 2.** Medical characteristics of the study participants.

Clinical data	N (%)
**Length of T2DM**	
Newly diagnosed	154 (33.5)
1–5 years	138 (3)
Between 5–10 years	88 (19.1)
Greater than 10 years	80 (17.4)
**Monitoring laboratory test value**	
3 months prior to data collection	170 (37)
6 months prior to data collection	110 (23.9)
One year prior to data collection	127 (27.6)
More than one year prior to data collection	53 (11.5)
**Medications**	
Oral pills	333 (72.4)
Insulin	75 (16.3)
Combinations	52 (11.3)
**T2DM complications**	
None	313 (68)
Neuropathy	26 (5.7)
Renal disease	26 (5.7)
Retinopathy	62 (13.5)
Foot disease	33 (7.2)
**Family history of T2DM**	
No	52 (11.3)
Father	143 (31.3)
Mother	56 (12.2)
Sisters and brothers	37 (8)
Relatives	44 (9.6)
More than one choice	128 (27.8)
**Co-morbidities**	
Coronary disorders	98 (21.3)
Osteoporosis	43 (9.3)
Hypertension	109 (23.7)
Kidney disorders	40 (8.7)
Anemia	84 (18.3)
Others ^a^	29 (12.7)

^a^ Retinal eye disease, nasal allergy, ear disease, liver disease, foot disease, polycystic ovary syndrome, thyroid, asthma, gastrointestinal disorders, and thrombocytopenia.

### 3.3 Knowledge, attitudes, and practices concerning the dietary supplements use

***3.3.1 Knowledge***

**Figure 1.**
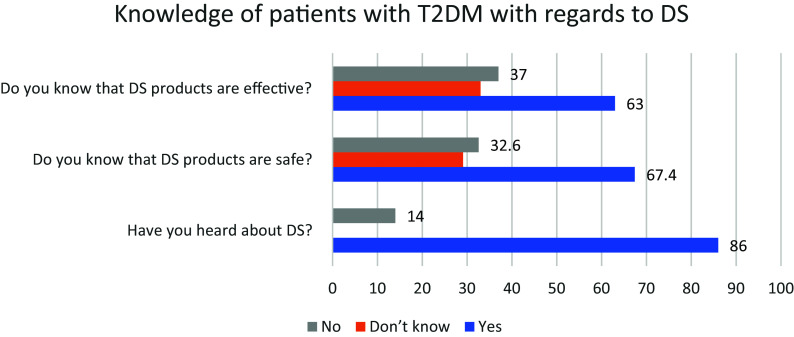
Participants’ responses to the knowledge questions concerning DS use. X axis refers to the percentage of patients responding the questions raised in Y axis. DS, Dietary supplements.

Figure 1 shows the participants’ knowledge towards the use of DS among persons with T2DM during the economic crisis. It appears that most of them (85.9%) had heard about DS, more than half of the sample (63%) knew that DS had efficacity, and 67% believed that DS are safe as shown in Figure 1.

***3.3.2 Attitudes***

**Figure 2.**
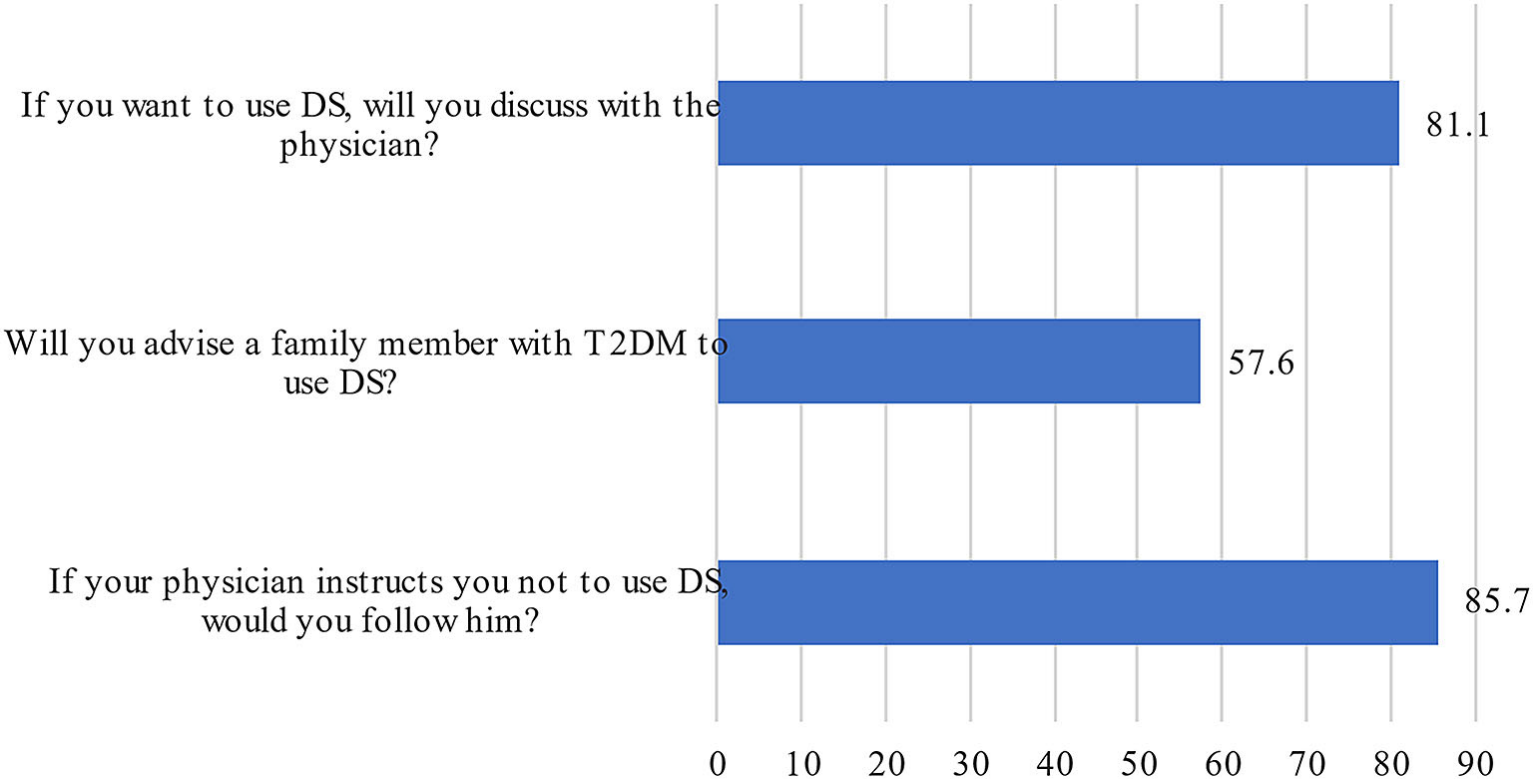
Participants’ responses to the attitude questions concerning DS use. X axis refers to the percentage of patients responding to the questions raised in Y axis. Only “Yes” responses are shown in this Figure. DS, Dietary supplements.

The participants’ attitudes regarding DS use are presented in Figure 2. It was shown that 81% of patients would first discuss DS use with their physician and that the majority (85.7%) would not use it if their physicians didn’t recommend it. On the other hand, more than half of participants (57.6%) suggested the same DS to a family member as shown in Figure 2. Table 3 displays the results of the participants’ attitudes based on a Likert scale scoring system consisting of responses of strongly agree, agree, neutral, disagree, and strongly disagree. “Strongly agree” and “agree” responses were combined to show the total percentage of “good attitude,” “neutral” for “not aware”, while “disagree” and “strongly disagree” were also combined to show the total percentage for “poor attitude.” One-third of the participants agreed that nutritional supplements can control the management of diabetes and prevent further complications such as retinal disease, foot disease, kidney disease, and nerve damage. On the contrary, only 34% of patients disagreed that nutritional supplements are a suitable substitute for a healthy and balanced diet in the treatment of diabetes. Furthermore, more than 44% disagreed that nutritional supplements are as effective as medication in modifying blood sugar levels in diabetic patients.

**Table 3.** Frequency of responses to the attitudes-related questions that are based on a Likert scale scoring system.

Attitude	Agree		Strongly agree		Neutral		Disagree		Strngly disagree	
	N	%	n	%	n	%	n	%	n	%
Are nutritional supplements or some of them necessary to control diabetes?	112	24.3	44	9.6	218	47.4	50	10.9	36	7.8
Do you think that nutritional supplements or some of them are a suitable alternative to a healthy and balanced diet for diabetics?	88	19.1	24	5.2	192	41.7	92	20.0	64	13.9
Do you think that DS prevents diabetes complications?	106	23.0	37	8.0	214	46.5	65	14.1	38	8.3
In your opinion, do nutritional supplements have the same effectiveness as medications in modifying the blood sugar level in diabetic patients?	61	13.3	29	6.3	168	36.5	89	19.3	113	24.6

***3.3.3 Practices***

**Figure 3.**
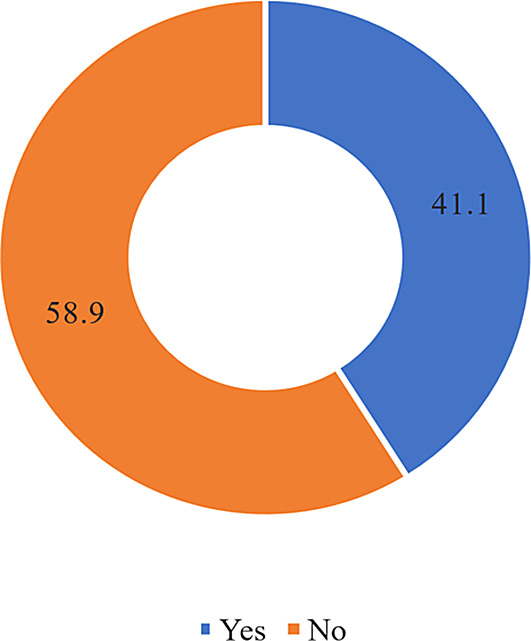
Prevalence responses of DS use and practices among patients with T2DM. DS, Dietary supplements; T2DM, type 2 diabetes mellitus.

**Figure 4.**
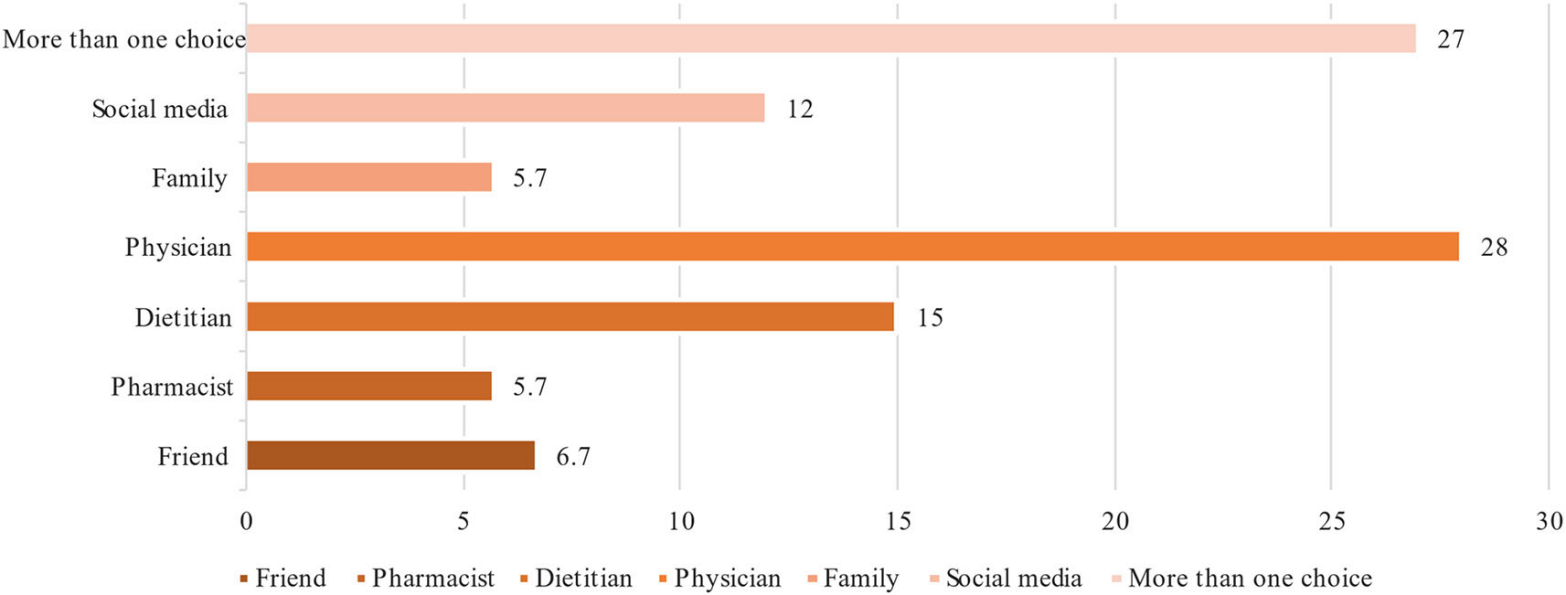
Sources of information on DS for patients with T2DM. DS, Dietary supplements; T2DM, type 2 diabetes mellitus.

Among people with T2DM, the estimated prevalence of DS use during the escalating crisis was 41.1% as shown in Figure 3. It appears that more than half the participants used previously DS to prevent other medical complications and combine DS with their diabetes medications. This use was always controlled by their physicians rather than using it on their own as shown in Figure 4. Multivitamins (27.6%) and vitamin C (22.4%) were the most DS used daily ( Table 4). Furthermore, per monthly and/or weekly use, vitamin D (11.3%), ginger supplements (14.3%) and green tea supplements (7.4%) were the most recorded DS ( Table 4). According to Table 5, around 41.1% of the participants complain about hypoglycemia and used DS to control their blood sugar levels (56.4%), while the rest used it to improve their health (35.5%) and control their diet (2.2%). What’s more, it has been demonstrated that 65% of the participants always read the DS label before ingesting it and 67.6% will use always continue using the DS ( Table 5).

**Table 4.** Frequency of consumption of dietary supplements by patients with T2DM.

DS practices	Everyday N (%)	1/week N (%)	1/month N (%)	Several times/week N (%)	Never N (%)
Multivitamins	127 (27.6)	25 (5.4)	16 (3.5)	17 (3.7)	275 (59.8)
Vitamin A	20 (4.3)	15 (3.3)	29 (6.3)	13 (2.8)	383 (83.3)
Vitamin C	103 (22.4)	32 (7)	33 (7.2)	30 (6.5)	262 (57)
Vitamin D	99 (21.5)	52 (11.3)	36 (7.8)	30 (6.5)	243 (52.8)
Vitamin E	10 (2.2)	17 (3.7)	35 (7.6)	10 (2.2)	388 (84.3)
Vitamin B1	13 (2.8)	17 (3.7)	21 (4.6)	14 (3)	395 (85.9)
Vitamin B2	11 (2.4)	10 (2.2)	23 (5)	15 (3.3)	401 (87.2)
Vitamin B3	10 (2.2)	14 (3)	26 (5.7)	11 (2.4)	399 (86.7)
Vitamin B6	38 (8.3)	19 (4.1)	26 (5.7)	18 (3.9)	359 (78)
Vitamin B12	48 (10.4)	22 (4.8)	31 (6.7)	13 (2.8)	346 (75.2)
Folic acid	23 (5)	11 (2.4)	27 (5.9)	12 (2.6)	387 (84.1)
Calcium	80 (17.4)	30 (6.5)	23 (5)	19 (4.1)	308 (67)
Chromium	11 (2.4)	18 (3.9)	29 (6.3)	7 (1.5)	395 (85.9)
Iron	83(18)	14 (3)	31 (6.7)	16 (3.5)	316 (68.7)
Magnesium	81 (17.6)	21 (4.6)	28 (6.1)	20 (4.3)	310 (67.4)
Zinc	37 (8)	15 (3.3)	29 (6.3)	13 (2.8)	366 (79.6)
Iodine	11 (2.4)	17 (3.7)	27 (5.9)	10 (2.2)	395 (85.9)
Cumin	43 (9.3)	31 (6.7)	37 (8)	25 (5.4)	324 (70.4)
Aloe vera	22 (4.8)	24 (5.2)	49 (10.7)	13 (2.8)	352 (76.5)
Ginger	64 (13.9)	34 (7.4)	66 (14.3)	28 (6.1)	268 (58.3)
Green tea	79 (17.2)	45 (9.8)	46 (10)	34 (7.4)	256 (55.7)

**Table 5.** Answers to questions on using dietary supplements, symptoms, labels, and continuous use.

Questions	N (%)
**The reason to use DS**	
To improve sugar level	259 (56.4)
To improve health	163 (35.5)
To improve the diet	10 (2.2)
Don’t use	27 (5.9)
**Symptoms due to DS use**	
None	258 (56.1)
Hypoglycemia	189 (41.1)
Tachycardia	1 (0.2)
Abdominal pain	5 (1.1)
Loss of appetite	1 (0.2)
Anxious	4 (0.9)
Headache	2 (0.4)
**Do you read the label of DS before using it**	
Always	298 (64.8)
Sometimes	131 (28.5)
Scarcely	8 (1.7)
Never	23 (5)
**Will you use nutritional supplements again?**	
Yes	311 (67.6)
No	149 (32.4)

### 3.4 Determinants of DS use using binary logistic analysis among study participants

Based on the bivariate analysis, we attempted to determine the extent of the contribution of the variables of interest to the probability of dietary supplements use among patients with T2DM using the logistic regression analysis. Table 6 shows that the gender, age, residency, marital status, monthly income, being employed, being on diet, having other comorbidities, length of diabetes disease, family history, knowledge about the efficacity and safety of DS and the source of information regarding DS were not considered as predictors for the use of DS among T2MD patients. On the other hand, the level of education can mediate the use of DS. For instance, those who are university graduates ranked first among DS consumers compared to those who are uneducated or above [OR=3.9, CI=1.5-10, p=0.003). In addition, patients with T2DM who monitor less their blood sugars have a 5 times higher odd to buy more DS compared to their counterparts (OR=4.3, CI=1.78-10.8; p=0.001). Furthermore, patients who never read the label were more prone to buy DS compared to patients who always read the label [OR=28.2, CI=3.5-225, p=0.002].

**Table 6.** Determinants of DS use among patients with T2DM.

Variables	DS use N	%	P value	OR	Lower	Upper
**Gender**			0.09			
Female	224	48.7				
Male	236	51.3		0.63	0.36	1.08
**Age**			0.12			
≤40	163	35.4				
41-49	68	14.8	0.04	0.46	0.21	0.99
50-59	142	30.9	0.79	0.91	0.47	1.77
≥ 60	87	18.9	0.17	0.55	0.24	1.28
**Address**			0.7			
Beirut and Mount Lebanon	76	16.5				
North Lebanon	352	76.5	0.33	1.35	0.73	2.50
Baalbek/Hermel and Beqaa	27	5.9	0.49	1.49	0.47	4.70
South Lebanon	5	1.1	0.40	2.95	0.22	38.55
**Education**			**0.024**			
Uneducated	76	16.5				
Primary level	65	14.1	**0.027**	3.17	1.14	8.86
Secondary level	64	13.9	0.46	1.43	0.55	3.73
High school level	72	15.7	0.16	1.97	0.75	5.16
University level	183	39.8	**0.003**	3.98	1.58	10.01
**Marital status**			0.39			
Single	113	24.6				
Married	277	60.2	0.10	1.67	0.89	3.14
Divorced	37	8.0	0.56	1.32	0.50	3.49
Widower	33	7.2	0.19	1.97	0.70	5.55
**Employment**			0.25			
Don’t work	190	41.3				
Have a job	270	58.7		1.61	0.71	3.65
**Income**			0.209			
No income	163	35.4				
Less than 100 $	153	33.3	0.139	0.53	0.23	1.22
Between 100$ and 300$	66	14.3	0.717	0.83	0.31	2.23
Greater than 300 $	78	17	0.097	0.42	0.15	1.16
**Other comorbidities**			0.679			
No	224	48.7				
Yes	236	51.3		0.90	0.55	1.46
**Adherence to diet**			0.622			
No	50	10.9				
Yes	164	35.7	0.765	1.17	0.40	3.38
Sometimes	246	53.5	0.711	0.82	0.29	2.30
**Duration of having diabetes**			0.802			
Newly diagnosed	154	33.5				
Less than 5 years	138	30.0	0.93	0.97	0.52	1.79
Between 5 and 15 years	127	27.6	0.47	0.78	0.39	1.53
Greater than 15 years	41	8.9	0.44	0.68	0.25	1.80
**Monitoring tests Laboratory values**			0.008*			
Since 3 months	170	37.0				
Since 6 months	110	23.9	0.76	0.91	0.49	1.68
Since 1 year	127	27.6	0.52	1.20	0.67	2.15
More than 1 year	53	11.5	**0.001**	4.39	1.78	10.82
**Family history**			0.879			
No	52	11.3				
Yes	408	88.7		0.94	0.43	2.0
**Heard DS**			0.88			
Yes	395	85.9				
No	65	14.1		1.06	0.45	2.5
**Safe DS**			0.12			
Yes	310	67.4				
No	16	3.5	0.17	3.99	0.55	28.9
Don’t know	134	29.1	0.09	1.86	0.90	3.8
**Effective DS**			0.41			
Yes	290	63.0				
No	18	3.9	0.37	0.48	0.10	2.3
Don’t know	152	33.0	0.44	1.31	0.65	2.6
**Source of information**			0.98			
Medical and paramedical	224	48.7				
Other	236	51.3		0.99	0.61	1.6
**Reading label**			**0.000**			
Always	298	64.8				
Sometimes	131	28.5	**0.000**	6.87	3.75	12.5
Scarcely	8	1.7	**0.056**	8.82	0.94	82.4
Never	23	5.0	**0.002**	28.26	3.53	225.8

## 4. Discussion

To the best of our knowledge, this is the first study that investigates the prevalence, and correlates of DS use among patients with T2DM during the economic crisis and drug shortage time in Lebanon. Almost 4 out of 10 patients with T2DM in our study were found to be using and abusing DS. Compared to the Unites states of America (USA), it was shown that 6 out of 10 Americans with T2DM use DS. ^15^ The prevalence reported in our study was higher than that reported in Saudi Arabia (17.6%), ^16^ Australia (24%), ^17^ and Thailand (5.3%) ^18^ but lower than the prevalence observed in Canada (44%), ^19^ and in 3 studies from the United States of America (USA) (62.1%; 67%; 58%). ^20^^–^^22^

The most common DS used daily in the current study were multivitamins (27.6%), vitamin C (22.4%), followed by vitamin D (21.5%), iron (18%), and calcium (17.4%). These findings came hand in hand with the data reported in a Canadian study ^19^ (27.5% multivitamins, 18.9% vitamin E, 18.7% vitamin C, and 16% calcium), but it contradicts the results reported in Clifford et al., where in Australia vitamin C (18%) ranked first followed by garlic (17%), omega 3 (14%), vitamin E (13%) and multivitamins (12%). ^17^

According to the literature, the use of DS for diabetes management has always been reviewed from a pharmacy standpoint and from that of complementary and alternative medicine. ^23^ However, as supplement use continues to grow in Lebanon, ^9^^–^^11^ it is important for healthcare professionals to understand the evidence behind prescribing supplements and their potential role as part of medical care especially during the unstable conditions. For instance, according to the literature review, meta-analyses assessing the impact of vitamin C supplementation on diabetes-related outcomes assumed an improvement of fasting blood glucose only without any improvement in HbA1c in patients with T2DM. ^24^^–^^26^ In addition, three meta-analyses that examined folate or folic acid supplementation had conflicting findings. ^27^^–^^29^ As for B12 supplementation, many studies showed that individuals taking Metformin suffer from depleted serum B12 levels, and human studies on both B6 and biotin were extremely limited, with a lack of narrative reviews on both vitamins’ impact on T2DM patients. ^30^ On the other hand, a meta-analysis that assessed the niacin supplementation showed an increased risk of T2DM onset following supplementation. ^31^ As for vitamin D, a review conducted by Li et al. showed that most studies in patients with T2DM used vitamin D at 2000 IU/day, which may improve glycemic control and a dose of 4000 IU/day may be elicited to provide positive effects on HbA1c, HOMA-IR, and the fasting plasma glucose. ^32^

The DS used by our study population was prescribed mainly by their physicians (70.2%). However, in the Arabian Gulf states, patients with T2DM did not reveal, discuss, or even seek medical advice from a physician, which differed from our findings, where participants relied heavily on physicians for DS advice. For instance, out of six studies conducted in the Gulf Club Countries (GCC), most DS users did not tell their physicians about the use of DS. ^16^^,^^33^^–^^37^ Similarly, in Nigeria and the USA where the majority of DS among patients with T2DM were not being taken based on a recommendation from a health provider. ^15^^,^^38^

More than half of the participants in our study were satisfied with their DS use and intended to use it again (67.6%). Unlike Saudi Arabian participants with T2DM, where only 6.7% of respondents said they would use DS again, and 55.7% regretted its use. ^14^ In our study, 85% of the participants had heard of DS, some of them (67.4%) believed DS were safe, and more than half (63%) knew that DS are effective where 33.9% agreed that DS are necessary to control diabetes and 31% strongly agreed that DS prevent diabetic complications. These findings align with the data reported in an updated literature review that showed that patients usually expressed the attitude that DS may not help much but will not hurt. ^39^ Additionally, our participants’ main reason for utilizing DS in this study was to lower their blood sugar level (56.1%), followed by improving overall health (35.5%). Thus, a responsible healthcare approach is much needed for the patients to receive evidence-based DS information about efficacy, effectiveness, adverse effects, and possible interactions. A slight majority (57.2%) of participants supported the use of DS in conjunction with their medical treatment for T2DM. This finding aligns with the result observed in a Saudi Arabian and Nigerian study where 90% and 67% of the patients with T2DM preferred combining DS with their conventional therapies, respectively. ^16^^,^^38^

In a qualitative study in Pakistan, the principal motivator of DS use was the desire to cure T2DM, where 41% preferred combining DS and T2DM. ^40^

In our study, the level of education, the frequency of monitoring blood sugars, and reading labels can mediate the use of DS. On contrary, the predictors of DS use in Saudi Arabia were age above 51 years, unemployment, and the participants’ knowledge about the effectiveness of complementary and alternative medicine (CAM) products. ^16^ Moreover, in Thailand, female gender, age 40-69 years, and diabetes duration of less than 10 years were significant correlators of DS use. ^41^ A Chinese study found that DS use among people with T2DM was associated with a history of previous DS use for other conditions, a positive attitude towards DS, efficacy of DS, and a longer duration of diabetes. ^42^

Moreover, a study from Malaysia found that females were 1.8 times more likely to use DS than males. ^43^ Furthermore, a study from Bahrain showed that females DS users were more likely to be dominating, and those who have had diabetes for a longer time and have T2DM complications were the top users. ^44^ Another study from Saudi Arabia showed that the most common users of DS practices were older females, housewives, and illiterates. ^45^

### Clinical implications of the current study

The current study can provide valuable insights for healthcare professionals to optimize patient care and support informed decision-making regarding supplement use in several ways:

1. Improved patient education: Understanding the knowledge, attitudes, and practices of dietary supplement use among individuals with diabetes can help healthcare professionals tailor their patient education efforts. They can provide more targeted information on the benefits and risks of specific supplements, as well as guidance on safe and effective supplement use.
2. Enhanced treatment planning: Healthcare professionals can use the findings from the study to inform their treatment planning for individuals with diabetes. They can incorporate evidence-based recommendations on dietary supplements that may complement conventional treatment approaches and support overall health outcomes.
3. Identification of gaps in care: The study can help healthcare professionals identify any gaps in care related to dietary supplement use among individuals with diabetes. This information can guide the development of interventions or resources to address these gaps and improve patient outcomes.
4. Promotion of evidence-based practice: By understanding the knowledge, attitudes, and practices of dietary supplement use among individuals with diabetes, healthcare professionals can promote evidence-based practice and discourage the use of potentially harmful or ineffective supplements. This can lead to better health outcomes for individuals with diabetes.

### Limits and strengths

This study presents some limitations. First, it lacks the impact of DS on T2DM compared to conventional therapy. Second, a self-reported questionnaire was used for most of the reported measures. Thus, bias may be present due to inaccurate self-reporting and memory in some questions. Third, this study was of cross-sectional survey; therefore, only associations can be determined and not causations. Notably, the strength of this study is that it is the first study that has been carried out in Lebanon which brought up the topic of knowledge, attitudes, and practices to DS use among patients with T2DM.

## 5. Conclusion

This study estimated the prevalence of DS use and abuse among patients with T2DM during the time of medicine shortage and economic crisis. Public health experts should encourage healthy discussions with their patients to comprehend their views regarding DS use. In addition, clinicians and researchers should collaborate to initiate safety and efficacy trials on common DS used for diabetes. Accordingly, relevant institutions whether governmental and non-governmental organizations, are strongly asked to design awareness programs that will be addressed to target groups and implemented by a specialized team in which social workers and health promoters play an important role by developing materials which fit all the perception of all categories that were shown by the study, and especially suitable to those who are illiterate, and who cannot read labels. The presence of evidence-based studies in the form of randomized controlled trials will help both patients and clinicians regarding the use of a DS product.

## Ethics and consent

The study was approved by the ethical committee of al Zahraa University Medical Center (#157/May 7, 2022). Written informed consent was obtained from all subjects involved in the study. Written informed consent was obtained also from the patient(s) to publish this paper.

## Authors’ contributions

MA, RM and MH conceived the idea, designed the study, helped in collecting the data, analyzed the data, drafted and reviewed the manuscript. SD helped in collecting the data, reviewed and edited the manuscript. NA and MS collected and analyzed the data. All authors read and approved the final manuscript.

## Data Availability

Open Science Framework: Dietary Supplement and Diabetes type II. https://doi.org/10.17605/OSF.IO/ZVNJD. ^46^ This project contains the following underlying data
- Dietary supplement and diabetes spss.sav - STROBE checklist Data are available under the terms of the Creative Commons Zero “No rights reserved” data waiver (CC0 1.0 Public domain dedication).
