# Supplementary material for: Knowledge, attitudes, and practices of Lebanese patients with type II diabetes towards the use and abuse of dietary supplements: A cross-sectional study
Source: F1000Res. 2024 Jul 26;13:432. Originally published 2024 May 1. [Version 2] doi: 10.12688/f1000research.146998.2 (PMC11301128; doi:10.12688/f1000research.146998.2)
Supplement: Peer review history

# Reviewer report: Reviewer response for version 2

**DOI:** 10.5256/f1000research.168897.r307414

**Published:** 2024-08-05

**Authors:** Abeer Salman Alzaben

**Affiliations:** 1 Department of Health Sciences, Princess Nourah bint Abdulrahman University, Riyadh, Riyadh Province, Saudi Arabia

Thank you for making the required changes.

Is the work clearly and accurately presented and does it cite the current literature?

Partly

If applicable, is the statistical analysis and its interpretation appropriate?

I cannot comment. A qualified statistician is required.

Are all the source data underlying the results available to ensure full reproducibility?

No source data required

Is the study design appropriate and is the work technically sound?

Partly

Are the conclusions drawn adequately supported by the results?

Partly

Are sufficient details of methods and analysis provided to allow replication by others?

Yes

Reviewer Expertise:

NA

I confirm that I have read this submission and believe that I have an appropriate level of expertise to confirm that it is of an acceptable scientific standard.

# Reviewer report: Reviewer response for version 2

**DOI:** 10.5256/f1000research.168897.r307415

**Published:** 2024-07-27

**Authors:** Rabih Hallit

**Affiliations:** 1 Holy Spirit University of Kaslik, Jounieh, Lebanon

Dear authors,

 thank you for the changes made. No more comments from my side. Good luck!

Is the work clearly and accurately presented and does it cite the current literature?

Partly

If applicable, is the statistical analysis and its interpretation appropriate?

Partly

Are all the source data underlying the results available to ensure full reproducibility?

Yes

Is the study design appropriate and is the work technically sound?

Yes

Are the conclusions drawn adequately supported by the results?

Yes

Are sufficient details of methods and analysis provided to allow replication by others?

Yes

Reviewer Expertise:

Infectious disease and internal medicine

I confirm that I have read this submission and believe that I have an appropriate level of expertise to confirm that it is of an acceptable scientific standard.

# Reviewer report: Reviewer response for version 1

**DOI:** 10.5256/f1000research.161131.r275467

**Published:** 2024-06-21

**Authors:** Abeer Salman Alzaben

**Affiliations:** 1 Department of Health Sciences, Princess Nourah bint Abdulrahman University, Riyadh, Riyadh Province, Saudi Arabia

Thank you for  providing  me the opportunity to review the manuscript. below some of my comments to make the manuscript more clear:

 Introduction :

 ·      Introduction is too short and lack of the rational. If there is shortage in medicine in Lebanon. why the authors worry about DS?  

 . may be need some information about % Dm in Lebanon or DS use ?

 ·      Does the objective to assess DS among all individuals with DM or only Type 2?

  Methods :

 ·      Please correct “convenient sample size was collected from all governorates in Lebanon. A total of 460 patients with T2DM were enrolled in this survey to “convenient sample technique”

 Inclusion/ exclusion criteria, 

 ·      “The exclusion criteria were individuals under the age of 18 years because of the high probability of having type 1 diabetes mellitus at this age.” Some individuals maybe older than 18 with type 1. Are those excluded? 

 ·      What about other disease such as Celiac or food allergies. Are those excluded?

 ·       Regarding the KSP. More information is needed about scoring. does the questions with one option 

 ·       Regarding the use of DS, I feel the questions/information are not clear. Do you mean they are currently use? Past use? If past use, how frequent? If a person has taken 1 pill in the last year. How you consider this? Need more clarifications.

 Results : 

 ·       Table 1: You had 14 % illiterate. Do you think those are significantly different in KSP and DS use than others? 

 ·       Duration of Disease newly diagnosis vs < 5 years.  I would say 1- 5 years are not newly diagnosis. Can you separate them as one subgroup? 

 ·        For me, it is still not clear, all sample with type 2 DM?  Did you exclude type 1? 

 ·        Figure 1, x axis refers to % but the sum does not = 100%, have you heard about DS is = 86 %? Figure 1  there are 2 option yes and don’t know what about no ? In addition, the knowledge items that have been asked are not knowledge questions, maybe awareness?

 ·        Be consistent with decimals  

 ·       Figure 2 not clear. what does the bar stand for? I think you need to work on all your figure legends. explain what X and Y axis means, it refers to n number or %.

 ·        How many participants have never used DS? 

 ·       Table 6 in education, I did not see the illiterate group in the Determinants.

Is the work clearly and accurately presented and does it cite the current literature?

Partly

If applicable, is the statistical analysis and its interpretation appropriate?

I cannot comment. A qualified statistician is required.

Are all the source data underlying the results available to ensure full reproducibility?

No source data required

Is the study design appropriate and is the work technically sound?

Partly

Are the conclusions drawn adequately supported by the results?

Partly

Are sufficient details of methods and analysis provided to allow replication by others?

Yes

Reviewer Expertise:

Nutrition

I confirm that I have read this submission and believe that I have an appropriate level of expertise to confirm that it is of an acceptable scientific standard, however I have significant reservations, as outlined above.

# Response

**Published:** 2024-07-02

**Authors:** Maha Hoteit

**Affiliations:** Lebanese University, Lebanon

Introduction :

 - Introduction is too short and lack of the rational. If there is shortage in medicine in Lebanon. why the authors worry about DS?  

 Thank you for this comment. The introduction was amended based on your comments. We are worried about DS use because Lebanese patients with type 2 diabetes mellitus (T2DM) are experiencing medicine shortages with rise in medication prices, which leads the patients to be incapable of buying their medical drugs. This deviation increased the health risk for these patients and increased the use and abuse of dietary supplements (DS). This increase may be due to several factors, including patients' perceptions that DS's natural products are safer, more effective, and cheaper than conventional medicines. This was mentioned in lines 56-60.

 -May be need some information about % Dm in Lebanon or DS use ?

 There is no information concerning DS use among patients with diabetes type 2 in Lebanon but we already mentioned that we already assessed DS use among: children under 5 and their mothers, adults and athletes. This was added in lines 62-64.

 - Does the objective to assess DS among all individuals with DM or only Type 2?

 This is mentioned in rationale, objectives and methodology: patients with type 2 diabetes only.

  -Methods :

 - Please correct “convenient sample size was collected from all governorates in Lebanon. A total of 460 patients with T2DM were enrolled in this survey to “convenient sample technique”

 The whole paragraph was amended.

 Inclusion/ exclusion criteria, 

 “The exclusion criteria were individuals under the age of 18 years because of the high probability of having type 1 diabetes mellitus at this age.” Some individuals maybe older than 18 with type 1. Are those excluded? 

 Yes these patients were also excluded. This was amended in the same paragraph.

 What about other diseases such as Celiac or food allergies. Are those excluded?

 This was not mentioned as an exclusion criterion. However, none of our patients were having these diseases because we were collecting the medical history of the patients. All patients who were excluded were having abnormal levels of glycemia, confirmed having type 2 diabetes and not diabetes type 1.

 Regarding the KSP. More information is needed about scoring. does the questions with one option.

 As mentioned in the “Methodology” section, the questions were having multiple responses for instance the Table 3 displays the results of the participants’ attitudes based on a Likert scale scoring system consisting of responses of strongly agree, agree, neutral, disagree, and strongly disagree. There was no single score, but we were showing the response of the patients. We are planning in future studies to validate this score.

 Regarding the use of DS, I feel the questions/information are not clear. Do you mean they are currently use? Past use? If past use, how frequent? If a person has taken 1 pill in the last year. How you consider this? Need more clarifications.

 We already mention in “Methodology” that we are assessing the current use of the DS by the patients. The answers of these questions are mentioned in Table 4 showing the frequency of the current use by the patients (per day, week, month…). The use of pills was most considered compared to the quantity. Based on that the frequency was our main objective.

 Results : 

 Table 1: You had 14 % illiterate. Do you think those are significantly different in KSP and DS use than others? 

 This was presented in Table 6 which shows that people who are educated had an 1.4-3.9 higher odd of using DS compared to those who are illiterate.

 ·       Duration of Disease newly diagnosis vs < 5 years.  I would say 1- 5 years are not newly diagnosis. Can you separate them as one subgroup? 

 Newly diagnosed are those who were diagnosed in the same year. The other sub-groups are defined as 1-5 years. This was amended in the manuscript and the tables.

 ·        For me, it is still not clear, all sample with type 2 DM?  Did you exclude type 1? 

 All patients with DT1 were excluded. And yes only patients with type 2 DM were included in the study.

 ·        Figure 1, x axis refers to % but the sum does not = 100%, have you heard about DS is = 86 %?

 86% of participants know and heard about DS and 14% don’t know about it. We only highlighted the answer “Yes” and “Don’t Know”. However, we amended Figure 1.

 Figure 1  there are 2 option yes and don’t know what about no ? In addition, the knowledge items that have been asked are not knowledge questions, maybe awareness?

 Based on the study source of the questionnaire, these questions were related to knowledge questions.

 ·        Be consistent with decimals  

 This was amended in the manuscript.

 ·       Figure 2 not clear. what does the bar stand for? I think you need to work on all your figure legends. explain what X and Y axis means, it refers to n number or %.

*X axis refers to the percentage of patients responding to the questions raised in Y axis. Only “Yes” responses are shown in this Figure. * This sentence was added to the Figures in the manuscript to make it more clearer.

 ·        How many participants have never used DS? 

 59% of the population in the current study. This is shown in Figure 3.

 ·       Table 6 in education, I did not see the illiterate group in the Determinants.

 It is available but said “ uneducated”. We will change it to be consistent.

# Reviewer report: Reviewer response for version 1

**DOI:** 10.5256/f1000research.161131.r275466

**Published:** 2024-05-10

**Authors:** Rabih Hallit

**Affiliations:** 1 Holy Spirit University of Kaslik, Jounieh, Lebanon

Thank you for inviting me to review this paper.

 Dear authors,

 Thank you for this paper. I have some comments that you might want to take into account in order to improve its quality:

 -The introduction is too short and can be easily expanded. You did not introduce the idea of dietary supplements, the knowledge, attitude and practice about these supplements according to previous international studies.

 -Rationale is missing: why is it important to conduct this study in patients with diabetes? The fact that the same study was conducted among patients with other diseases is not sufficient.

 -Your first objective needs to be introduced. What is the relation of the economic crisis in Lebanon with your study and with the use of dietary supplements in patients with diabetes?

 -In section 2.3, how did you build the questionnaire? based on previous studies or you created your own scale? I suggest adding the questionnaire as an appendix with the right answers for the knowledge questions most importantly.

 -The results lack the bivariate analyses. Please add them and then you enter in the multivariable model all variables that showed a p < 0.25 in the bivariate analysis only.

 -The discussion is good but it needs adjustment maybe after implementing the changes to the results.

 -Please add the clinical implications paragraph to the discussion: how would other healthcare professionals benefit from your study?

Is the work clearly and accurately presented and does it cite the current literature?

Partly

If applicable, is the statistical analysis and its interpretation appropriate?

Partly

Are all the source data underlying the results available to ensure full reproducibility?

Yes

Is the study design appropriate and is the work technically sound?

Yes

Are the conclusions drawn adequately supported by the results?

Yes

Are sufficient details of methods and analysis provided to allow replication by others?

Yes

Reviewer Expertise:

Infectious disease and internal medicine

I confirm that I have read this submission and believe that I have an appropriate level of expertise to confirm that it is of an acceptable scientific standard, however I have significant reservations, as outlined above.

# Response

**Published:** 2024-05-14

**Authors:** Maha Hoteit

**Affiliations:** Lebanese University, Lebanon

Thank you for inviting me to review this paper.

 Dear authors,

 Thank you for this paper. I have some comments that you might want to take into account in order to improve its quality:

 -The introduction is too short and can be easily expanded. You did not introduce the idea of dietary supplements, the knowledge, attitude and practice about these supplements according to previous international studies.

***Author Response:*** Done

 -Rationale is missing: why is it important to conduct this study in patients with diabetes? The fact that the same study was conducted among patients with other diseases is not sufficient.

***Author Response: *** Done

 -Your first objective needs to be introduced. What is the relation of the economic crisis in Lebanon with your study and with the use of dietary supplements in patients with diabetes?

***Author Response: *** Done

 -In section 2.3, how did you build the questionnaire? based on previous studies or you created your own scale? I suggest adding the questionnaire as an appendix with the right answers for the knowledge questions most importantly.

***Author Response: *** It was built based on previous published studies with no major changes and all the tools are already available on: Hoteit M: Dietary Supplement and Diabetes type II. [Dataset]. *OSF.* 2024. Publisher Full Text (reference 44 in the manuscript).

 As for the responses to knowledge, attitudes and practices, they are already mentioned in the Figures and tables in the manuscript itself.

 -The results lack the bivariate analyses. Please add them and then you enter in the multivariable model all variables that showed a p < 0.25 in the bivariate analysis only.

***Author Response: *** Bivariate analyses are already shown in Table 6.

 -The discussion is good but it needs adjustment maybe after implementing the changes to the results.

***Author Response: *** Done

 -Please add the clinical implications paragraph to the discussion: how would other healthcare professionals benefit from your study?

***Author Response: *** Done
